# Emerging Antibiotic Resistance to Bacterial Isolates from Human Urinary Tract Infections in Grenada

**DOI:** 10.7759/cureus.5752

**Published:** 2019-09-25

**Authors:** Deepak Sharma, Sara E Preston, Robert Hage

**Affiliations:** 1 Anatomy, St. George's University School of Medicine, St Georges, GRD; 2 Basic Science, St. George's University School of Medicine, St Georges, GRD; 3 Otolaryngology, St. George's University School of Medicine, St Georges, GRD

**Keywords:** bacterial isolates, antimicrobial drug resistance, urinary tract infection, grenada

## Abstract

A urinary tract infection (UTI) in humans is one of the most common ailments in developing countries. The treatment of UTI is becoming difficult because of the increasing drug resistance against the common bacteria associated with UTI. This research aimed to determine the bacteria, and their antimicrobial drug resistance, associated with UTI in the Grenada population. A retrospective study of data (2015 through 2017) from the microbiology laboratory of the Grenada General Hospital was analyzed. Bacteria were isolated from 1289 (33.3%) urine cultures of 3867 UTI suspected urine samples. Both Gram-positive (Staphylococcus aureus 5.0%; Enterococci group D 43.2%) and Gram-negative bacteria (Escherichia coli 51%; Klebsiella pneumoniae20.0%; Proteus mirabilis 10.0%; Acinetobacter spp. 20.0%) were isolated. Bacterial isolates were tested for their resistance to nine antibacterial drugs (ampicillin, gentamicin, norfloxacin, cefuroxime, ceftazidime, Bactrim, imipenem, augmentin, and ciprofloxacin). Gram-negative bacteria showed higher antimicrobial drug resistance.

## Introduction

Bacterial infections are a common cause of urinary disorders in humans and have become a common cause of nosocomial infections. Urinary tract infections (UTIs) accounted for 35% of hospitalized patients in Lafia, Nigeria, and 23% to 37% of patients attending general hospitals in Nepal [1-2].

The gold standard for diagnosing a UTI is the collection of mid-stream or catheter urine with a bacteriological culture [3]. Frequently isolated bacteria found in UTI are Escherichia coli (56.3%-77.3%) followed by Enterococcus faecalis, Klebsiella pneumoniae, Proteus spp., Acinetobacter spp., and Staphylococcus aureus [4-5]. The antibiotic resistance of the bacterial isolates of UTI is a challenge associated with higher mortality, morbidity, and cost [6]. Even though UTI is self-limiting, it is among the most common bacterial infections acquired in the community and in hospitals. A study in 2007 by Schappert and Rechtsteiner (2011) reported that 10.5 million patients presented annually with UTI symptoms during medical office visits in the United States [7]. With an estimated 154 million antibiotic prescriptions given across for all infections during 2010-2011 in the United States, an estimated 30% were inappropriate despite existing policies and guidelines for antibiotic use. The overprescription and misuse of antibiotics because of economic and social pressures may contribute to the increasing resistance [8].

The World Health Organization (WHO) launched the global National Action Plan (NAP) to combat antimicrobial resistance in 2015 [9]. The suggested guidelines included: 1) Improved awareness and understanding of antimicrobial resistance; 2) Robust medical professional training to strengthen knowledge of the use of antimicrobial agents; 3) Creation of global innovation to increase surveillance of the incidence of infection and non-commercial research; 4) Providing substantial investment in medicines, diagnostic tools, vaccines, and other interventions; 5) Establishing antimicrobial resistance reference laboratories and bacterial strain banks [10-11]. Hence, in response to the NAP, this study was conducted to obtain an overview of the bacteria involved in UTI in the population of Grenada and to establish the in vitro resistance pattern for the most commonly used antimicrobial drugs for the treatment of UTI in Grenada.

## Materials and methods

Grenada is located in the West Indies and has a population of 110,000. The Grenada General Hospital lab is the main facility in Grenada for testing urine samples. A retrospective study of all clinically diagnosed infections of UTI was conducted for the years 2015 through 2017, resulting in 3867 urine samples of patients (males and females). Samples were expected to be first void morning mid-stream, catheter stream, bag urine, suprapubic, or cystoscopy urine samples. A minimum of 10 ml of urine in a sterile container needed to be submitted to the bacteriology laboratory of the General Hospital within two hours of collection. The sample rejection criteria included urine taken from a Foley catheter tip, urine in broth medium, urine specimens received after two hours of collection without refrigeration, and samples collected in non-sterile containers.

Bacterial culture of urine samples

The General Hospital lab procedure for the culture of urine was inoculation of 0.001 ml of urine on chromogenic agar (Chrom-Uni-Select 4 medium; Bio-Rad, California, US) using a standard inoculation loop and incubated at 37°C for 18-24 hours. The Uni-Select 4 medium is a non-selective chromogenic agar medium for the isolation, differentiation, and enumeration of urinary tract pathogens. The Uni-Select 4 medium allows for the differentiation and immediate identification of Escherichia (E.) coli, Enterococcus spp., P. mirabilis, and the presumptive identification of some other urinary bacterial pathogens, in particular, the KESC group of Enterobacteria (Klebsiella (K.), Enterobacter, Serratia, and Citrobacter), and the PMP group (Proteus (P.)-Morgonella, Providencia). All the bacterial colonies were Gram stained. All Gram-negative isolates were inoculated into API-20E (Analytical Profile Index, BioMetrieux Inc. Durham, NC, US) test strips and incubated at 37°C for 24 hours for confirmation of E. coli, Acinetobacter spp., K. pneumoniae, and P. mirabilis. For Gram-positive cultures, a catalase test was performed. Catalase-positive samples were subjected to a coagulase test and a novobiocin disc to identify Staphylococcus (S.) aureus. API STAPH (Bio-Merieux Inc.) was done for the confirmation of Staphylococcus spp. while Streptococcus spp. was confirmed using API 20 strep. (Biomerieux Inc, NC). 

Antimicrobial resistance test

The antibiotic sensitivity, intermediate, or resistance test was performed using the Kirby-Bauer disk diffusion method described by Clinical Laboratory Standards Institute (CLSI, 2015) guidelines on chromogenic agar [12]. The antimicrobial disc used was the BD BBL Sensi-Disc (BD, NJ, US), which included ampicillin 10 mg, augmentin (amoxicillin/clavulanic acid ) 20 mg, Bactrim (trimethoprim/ sulfamethoxazole) 1.25 mg, cefuroxime 30 mg, gentamicin 120 mg, norfloxacin 10 mg, ceftazidime 30 mg, ciprofloxacin 5 mg, and imipenem 10 mg. The inhibition zones were interpreted based on CLSI guidelines. A chi-square analysis was performed to evaluate the level of change in microbe-specific antibiotic resistance over the three-year period.

## Results

Bacteria were isolated from 1289 (33.3%) urine cultures out of 3867 UTI-suspected urine samples. The isolated bacteria were Gram-positive (34.6%) (S. aureus and Group D Enterococcus) and Gram-negative (65.4%) (Acinetobacter spp., E. coli, K. pneumoniae, and P. mirabilis). Frequently isolated bacteria were E. coli (51.5%), followed by group D Enterococci (43.2%) and K. pneumoniae (20.0%). The result of bacterial isolation is presented in Table 1.

**Table 1:** Number of Gram-positive and Gram-negative bacterial isolates from UTI in the Grenada population UTI: urinary tract infection

	2015	2016	2017	Total	Percent
Gram-positive (34.6 %)					
Staphylococcus aureus	18	16	11	45	5.0
Group D Enterococci	126	190	85	401	43.2
Gram-negative (65.4 %)					
Escherichia coli	145	236	97	479	51.5
Acinetobacter spp.	38	24	20	82	9.0
Klebsiella pneumonia	56	101	29	186	20.0
Proteus mirabilis	29	52	15	96	10.0

**Figure 1:**
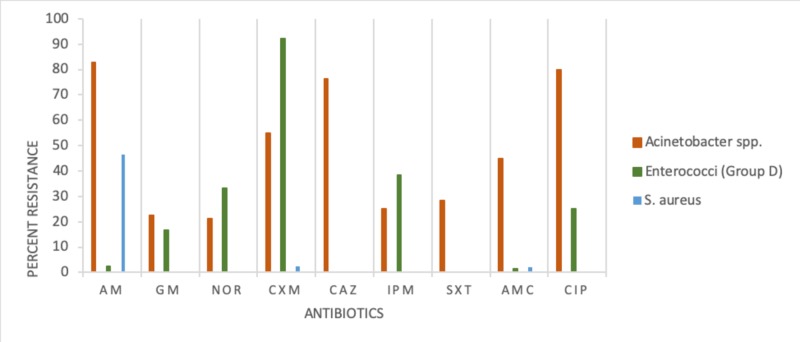
Antimicrobial resistance pattern for Acinetobacter spp., Enterococci (Group D), and S. aureus Index for antimicrobial drugs: AM-Ampicillin, GM-Gentamicin, NOR-Norfloxacin, CXM-Cefuroxime, CAZ-Ceftazidime, IPM-Imipenem, SXT-Bactrim, AMC-Augmentin, CIP-Ciprofloxacin

**Figure 2:**
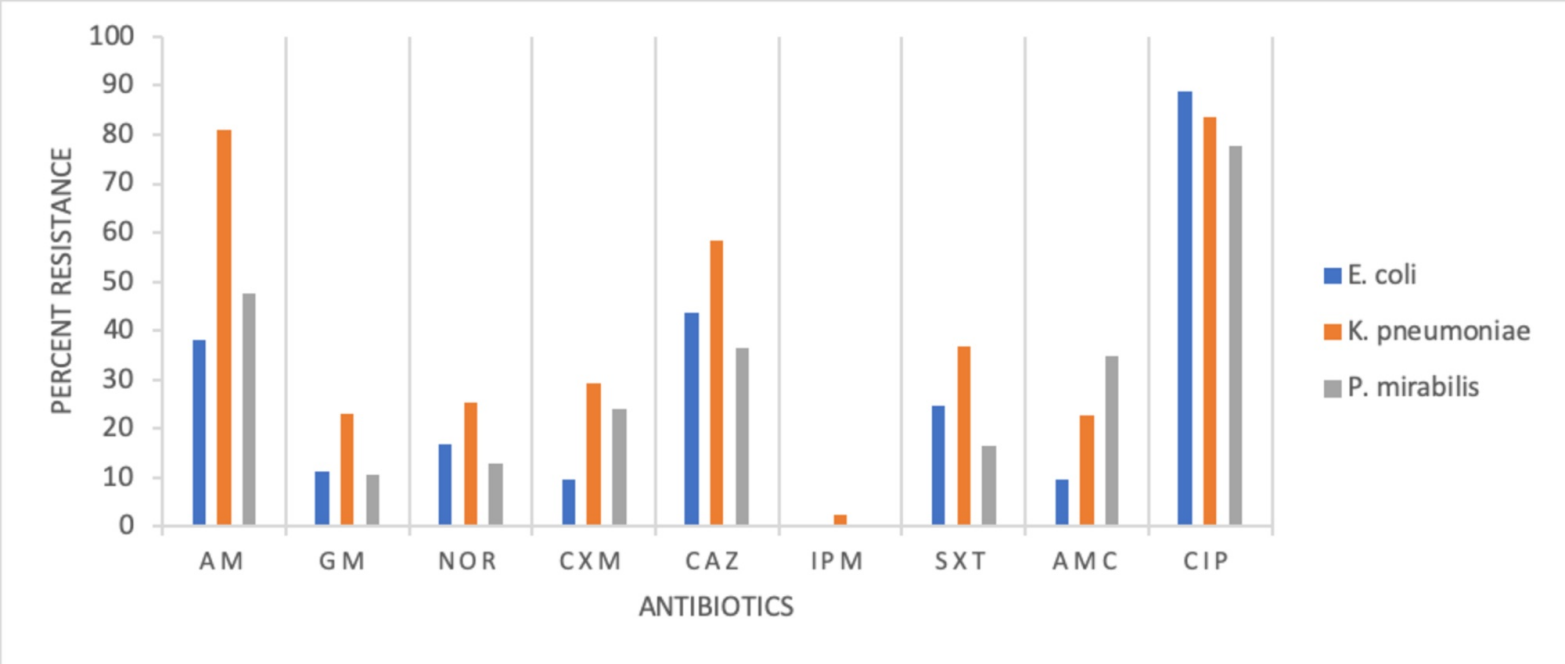
Antimicrobial resistance pattern for E. coli, K. pneumoniae, and P. mirabilis Index for antimicrobial drugs: AM-Ampicillin, GM-Gentamicin, NOR-Norfloxacin, CXM-Cefuroxime, CAZ-Ceftazidime, IPM-Imipenem, SXT-Bactrim, AMC-Augmentin, CIP-Ciprofloxacin

The result of the resistance pattern of bacterial isolates tested against nine antimicrobial drugs is presented in Figures 1-2. E. coli showed high resistance to six antimicrobial drugs in the following descending order: ampicillin, Bactrim, norfloxacin, gentamicin, augmentin, and cefuroxime. Enterobacter group D was resistant to cefuroxime (90.0%) and susceptible to the remaining antimicrobial drugs. K. pneumonia was resistant to eight drugs in the following descending order: ampicillin 80%, Bactrim 30%, cefuroxime 27%, and gentamicin, norfloxacin, augmentin, ciprofloxacin, and ceftazidime, ranging between 12% and 23%. S. aureus, a Gram-positive bacteria, was resistant to ampicillin (43%).

A chi-square analysis was employed to compare the bacterial isolates and antimicrobial resistance. The analysis showed no significant statistical difference in the resistance pattern among bacterial isolates (P>0.05).

## Discussion

Urine samples from 33.0% of UTI-suspected patients were positive for bacterial pathogens. Our finding coincides with Martin et al. (2019) who reported 32% positive urine samples from patients attending hospitals in Bushenie, Uganda, and Barton et al. (2008) who reported 38% positive urine samples from UTI in neonates from the University Hospital of the West Indies, Jamaica [13-14]. Higher numbers of bacterial pathogens in UTI samples were reported by Nerurkar et al. (2012) from western Mumbai, India (60%), Tia et al. (2016; 48%) from the Institute of Medical Science, Varanasi, India, and Stanley et al. (2014; 55%) from hospitals in Afikpo, Nigeria [15-17]. Lower numbers of bacterial isolates were found in UTI-suspected patients reported by Beyene et al. (2011; 9.2%) from Jimma University Specialized Hospital, Southwest Ethiopia, and Mahmoud et al. (2016; 13.9%) from Messalata Central Hospital, Libya and Mulugeta [8,18].

Our results indicate a higher number of Gram-negative bacterial isolates (65.5%) compared to Gram-positive isolates (34.6%). This finding is in agreement with Tia et al. (2016), Dinah et al. (2019), and Wariso et al. (2010) all of whom have reported more Gram-negative than Gram-positive isolates [11,16,19]. In our study, the highest number of isolates was for E. coli. This finding coincides with the majority of researchers, Dinah et al. (2019); Nerurkar et al. (2012); Tanja et al. (2011); Helen et al. (2018) [11,15,20-21].

In our study, the highest incidence of E. coli was followed by group D Enterococci, with similar results reported by Nerurkar et al. (2012) [15]. Studies by Stanley et al. (2014), Mahmoud et al. (2016), Beyene et al. (2011), Ganesh et al. (2019), and Dinah et al. (2019) reported that the incidence of E. coli was followed by K. pneumoniae [2,8,11,17-18]. Wariso et al. (2010) and Martin et al. (2019) reported the incidence of E.coli, followed by S. aureus isolates [13,19].

Our study recorded a varying degree of resistance to antimicrobial drugs. E. coli, K. pneumoniae, P. mirabilis, and Acinetobacter spp., all Gram-negative bacteria, showed high resistance to tested drugs against ampicillin, Bactrim, and augmentin. Whereas Gram-positive S. aureus was highly resistant to ampicillin (44%), similar to the incidence reported by Beyene et al. (2011) [18]. Gram-positive Acinetobacter spp. was highly resistant to ampicillin (83%), augmentin (46%), and ciprofloxacin(43%). Contrary to multiple drug resistance (E. coli, Acinetobacter spp., K. pneumoniae, and P. mirabilis), two pathogens showed single drug resistance, namely, Enterococci group D resistant to cefuroxime and S. aureus to ampicillin. The variation in bacterial isolate resistance pattern in cases of UTI studies is not completely explained.

Khan et al. (2015), Tia et al. (2016), and Tessema et al. (2007) suggested that variation in the pattern of antimicrobial drug resistance could be due to the social habits of the population and the volume of prior exposure of the same medication [16,22-23]. The recommended first-line treatment for a UTI infection in Grenada is trimethoprim/sulfamethoxazole (Bactrim) followed by ciprofloxacin and amoxicillin/clavulanate acid (augmentin). Therefore, the resistance pattern observed in our study could be due to the past promoted use of ciprofloxacin and the current recommended use of Bactrim and augmentin for most UTI infections in Grenada.

## Conclusions

Globally, studies have reported an emerging multi-drug resistance pattern in UTIs. Our study of UTI infections in Grenada, unique for the Caribbean, confirms that pattern. The results of our study contribute to the creation of a database that can be used for reporting and training and educating medical personnel. The responsible institutions can set up antibiotic stewardship to monitor the UTI drug-resistance pattern in the Caribbean region and provide guidelines for practicing medical personnel.
